# Exosomes and immune modulation: implications for neuroblastoma immunotherapy

**DOI:** 10.3389/fimmu.2025.1600062

**Published:** 2025-05-27

**Authors:** Martina Morini, Chiara Vitale, Martina Ardito, Alessandra Dondero, Katia Cortese, Cristina Bottino, Roberta Castriconi

**Affiliations:** ^1^ Laboratory of Experimental Therapies in Oncology, IRCCS Istituto Giannina Gaslini, Genova, Italy; ^2^ Department of Experimental Medicine, University of Genova, Genova, Italy; ^3^ Laboratory of Clinical and Experimental Immunology, IRCCS Istituto Giannina Gaslini, Genova, Italy

**Keywords:** exosomes, immunotherapy, tumor microenvironment, neuroblastoma, NK cells, cancer stem cells

## Abstract

Exosomes are nano-sized extracellular vesicles involved in cell homeostasis. Tumor-derived exosomes (TDEs) promote tumor progression by creating an immunosuppressive tumor microenvironment (TME), inhibiting T and NK cell activity, preventing dendritic cell maturation, and expanding immunosuppressive cell populations. Cancer Stem Cell (CSC)-derived exosomes further trigger functional changes in immune cells subsets, enhancing immune suppression. Consequently, blocking the release or the uptake of TDEs significantly impact immunotherapy efficacy, making them potential therapeutic targets. On the other hand, NK cell-derived exosomes can be engineered to carry immune-activating molecules or inhibitors of immune checkpoint molecules to elicit immune responses. This review highlights the interplay between TDEs and immune cells, particularly NK cells, in different tumors, with a focus on neuroblastoma, and explores exosome-based strategies to improve immunotherapy efficacy.

## 1 Introduction

Exosomes are nano-sized extracellular vesicles released by different cell types. They mediate intercellular communication by carrying a variety of biomolecules including proteins, lipids, and nucleic acids (1). Their function affects physiological and pathological processes, particularly in cancer biology. Tumor-derived exosomes (TDEs) interact with tumor and stromal tissues, reprogramming cells and facilitating tumor initiation and progression (2). Specifically, TDEs induce the establishment of an immunosuppressive tumor microenvironment (TME) by different mechanisms, including the suppression of T and Natural Killer (NK) cell activation and proliferation, the inhibition of maturation of Dendritic Cells (DCs), and the functional reprogramming of macrophages (3).

An immunosuppressive TME represent the main barrier to the efficacy of immunotherapeutic approaches that are often included in combined treatment against aggressive neoplastic diseases.

Neuroblastoma (NB) - the most common extracranial cancer diagnosed during infancy and originating from neural crest cells - has heterogeneous clinical manifestations, ranging from localized low risk disease to metastatic, high-risk (HR) tumors. The current therapeutic strategy for HR-NB patients includes immunotherapy with a monoclonal antibody targeting the disialoganglioside GD2, a marker expressed on NB cell surface (4). The crosstalk between tumor associated macrophages (TAMs) and NK cells play a major role in favoring tumor progression and immunotherapy resistance (5).

Moreover, solid tumors including NB are characterized by the presence of Cancer Stem Cells (CSCs), a rare cellular population with highly malignant features (6). CSCs release exosomes carrying stemness markers and regulators, which can trigger functional changes in immune cells, further contributing to the suppression of the immune response (7).

The multiple interactions occurring between tumor and immune cells via TDEs bring forward the impact of the released vesicles on the efficacy of immunotherapies. Reducing the immune suppression by blocking the release or the uptake of TDEs could have therapeutic benefits. A deeper knowledge of TDEs-mediated effects on the immune system would allow us to develop new treatments.

The present review will discuss the most relevant results on the crosstalk between TDEs and immune cells with a focus on NB. We will report how such interaction can shape the TME and, consequently, the response to immunotherapy-based treatments and how exosomes could be employed for developing novel therapeutic strategies aimed at improving immunotherapy efficacy.

## 2 The role of tumor-derived exosomes in immune suppression

**Figure 1:**
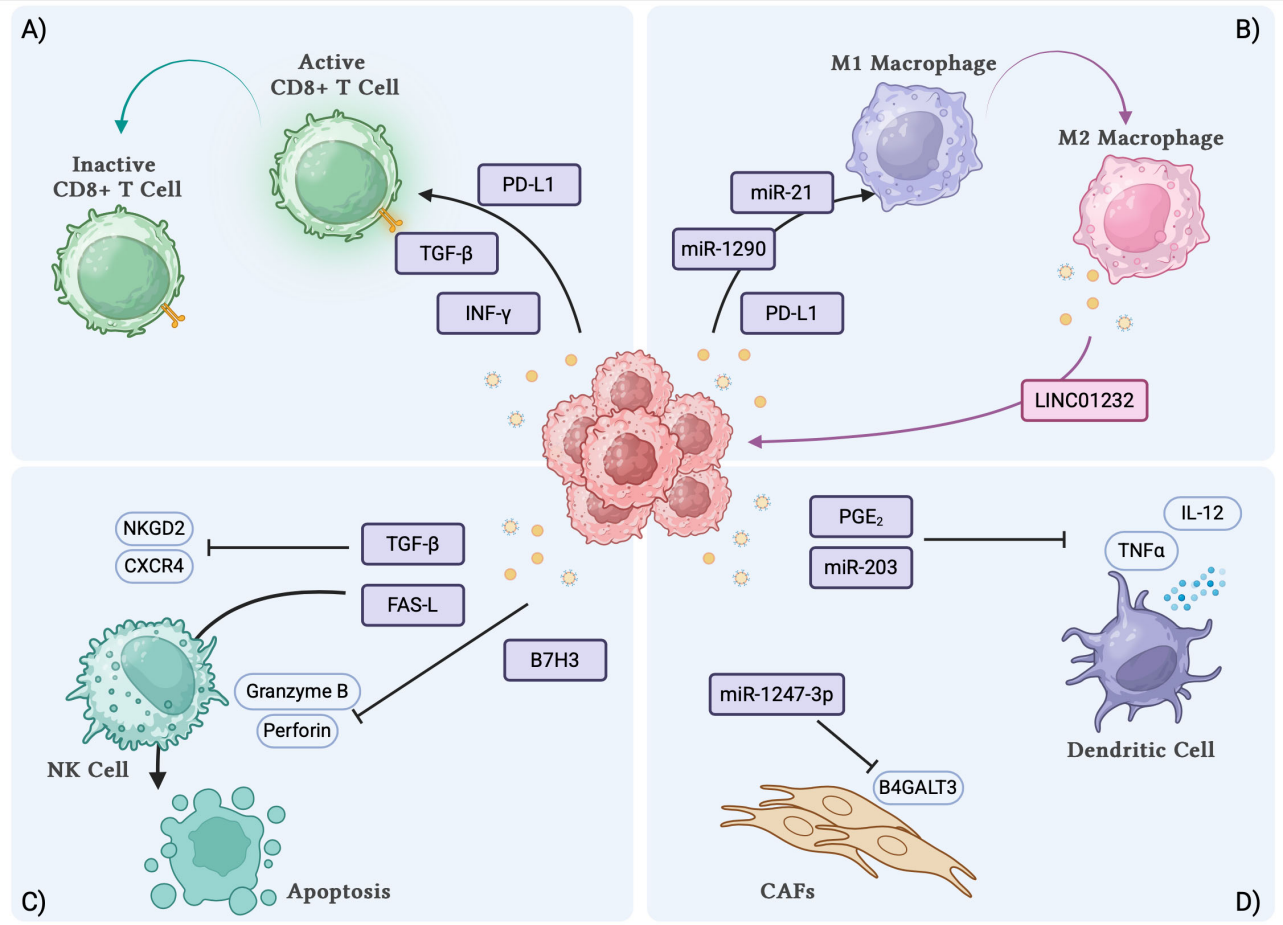
TDEs interactions with immune cell subsets and biological effects. **(A)** TDEs induce the inactivation of CD8+ T cells. **(B)** TDEs induce the polarization of macrophages towards M2 phenotype. **(C)** TDEs can inhibit the activation and induce the apoptosis of NK cells. **(D)** TDEs inhibit the secretory function of DCs and induce the transformation of fibroblasts to CAFs. TDEs, tumor derived exosomes; DCs, dendritic cells; CAFs, cancer associated fibroblasts. Created with BioRender.com.

Different components of the TME, including endothelial cells, extracellular matrix, fibroblasts, and immune cells, strongly influence cancer initiation and progression. The crosstalk between these components and tumor cells occurs mainly through the secretion of specific molecules exerting pro or antitumor activity. Over the last years, it has been shown that cellular communication occurs also through the release of TDEs, and extracellular vesicles, especially exosomes, that help cancer cells communicate also with distant cells. TDEs promote tumor growth and metastasis but also shape an immunosuppressive TME interfering with the activity of different immune cell populations (**Figure 1**).

### 2.1 TDEs modulate T cell immunity and macrophage function through PD-L1 and TGF-β

TDEs can suppress T cell activation and proliferation by carrying molecules such as programmed death-ligand 1 (PD-L1), transforming growth factor-β (TGF-β), and specific microRNAs. PD-L1 is a checkpoint molecule that binds to PD-1 on T cells, preventing their activation and enabling immune evasion. It has been observed in metastatic melanoma that exosomes carry surface PD-L1 (exo-PD-L1). Its expression is upregulated by Interferon-γ (IFN-γ), and in patients the levels of circulating exo-PD-L1 positively correlated with IFN-γ expression (8). Exo-PD-L1 was shown to inhibit the proliferation and the cytotoxic activity of CD8 T cells, and higher levels of exo-PD-L1 were associated with poorer response to treatment and clinical outcomes. High levels of exo-PD-L1 in pre-treated melanoma patients may reflect the exhaustion of T cells, making anti-PD1 therapy ineffective. For on-treatment patients, exo-PD-L1 levels increased early during treatment, correlating with T-cell reinvigoration and better clinical outcomes. This could reflect the attempt of tumor cells to inactivate CD8 T cells, which is though prevented by the ongoing anti-PD1 treatment that block the PD-L1/PD1 axis (8). These findings suggest that exo-PD-L1 may serve as a potential biomarker for melanoma progression and response to immunotherapy.

Within the TME, tumor-associated macrophages (TAMs) also express PD-L1, allowing them to contribute to anti-tumor response impairment. Exosomes mediate the crosstalk between TAMs and tumor cells, favoring tumor growth and progression. TDEs can induce TAMs to polarize towards the M2 phenotype, which is associated with increased PD-L1 expression and the release of exosomes inducing PD-L1 expression in tumor cells, a phenomenon mediated by exosomal molecules including microRNAs and cytokines (9). In gliomas, M2-polarized TAMs release exosomes enriched with LINC01232, a long non-coding RNA (lncRNA) that interacts with E2F2, promoting its nuclear entry and the subsequent transcription of the autophagy transport receptor NBR1 (10). This causes an increased degradation of MHC-I, reducing MHC-I expression on the tumor cell surface, and enabling the evasion from CD8+ T cell-mediated recognition.

The plasma of patients with head and neck squamous cell carcinoma is characterized by the presence of exosomes containing TGF-β (11). Importantly, the exosome concentration of exosome-associated TGF-β (exo-TGF-β) reflects tumor size and decreases after therapy. These findings suggest the potential use of exosome-associated TGF-β as a biomarker. Importantly, exo-TGF-β are also involved in different pro-tumor pathways including angiogenesis (11), the formation of cancer-associated fibroblasts (CAF) (12), and the initiation of the pre-metastatic niche formation (13). It has also been reported that exo-TGF-β can drive the remodeling of the pulmonary vascular niche, facilitating the lung colonization of triple-negative breast cancer cells (14). In pancreatic cancer cells, TGF-β mRNA is transferred via exosomes in recipient cancer cells, leading to increased TGF-β protein and activation of epithelial to mesenchymal transition (EMT), promoting early-stage metastasis (15).

### 2.2 TDEs carry immunomodulatory non-coding RNAs

#### 2.2.1 Exosomal microRNAs

TDEs immunomodulatory function is achieved also through the transfer of specific microRNAs, small non-coding RNA molecules regulating protein translation. The exosomal miR-1290 has been identified as a key player in antitumor immunity. Hypoxic lung adenocarcinoma cells release exosomes containing high levels of miR-1290, which targets and inhibits the suppressor of cytokine signaling 3 (SOCS3) (16). SOCS3 is involved in the maintenance of M1-type macrophages, thus its inhibition promotes the polarization toward the M2 phenotype. In hypoxic hepatocellular carcinoma, exo-miR-1290 induces the M2 polarization of macrophages by inhibiting Akt2 and upregulating PD-L1, stimulating CD8+ T cells apoptosis and triggering the EMT process, which promotes metastasis formation (17). In hepatoblastoma, the most frequent pediatric liver cancer, miR-126 delivered via exosomes promotes the differentiation of bone marrow mesenchymal stem cells into cancer stem cells, sustaining cancer growth, invasion and metastasis (18). Ewing sarcoma (EWs) cells expressing IGF2BP3 protein can release extracellular vesicles with a specific miRNA cargo that can positively regulate the migration rate of recipient cells, promoting metastasis formation (19). Moreover, EWs cells in hypoxic conditions produce miR-210-enriched exosomes, which downregulates the pro-apoptotic protein CASP8AP2, thus inhibiting apoptosis in recipient cancer cells (20).

The pro-metastatic effect of exosomes has been also reported in rhabdomyosarcoma (RMS), a rare soft tissue cancer affecting children. Exosomes released by metastatic RMS carry the transmembrane protein CD147, which facilitates the crosstalk between tumor and stromal cells, enhancing tumor invasive properties and modulating the TME (21).

MiR-21 is also known for its oncogenic function in different cancer types, including non-small cell lung cancer, breast cancer, and head and neck squamous cell carcinoma (HNSCC) (22–24). HNSCC cancer cells overexpress miR-21 and release TDEs abundant in miR-21. Such miR-21-enriched TDEs are taken up by TAMs that polarize toward M2, favoring the establishment of an immunosuppressive TME (25). TDEs enriched in miR-21 have also been detected in osteosarcoma (OS), a malignant pediatric tumor of the bone. Exosomes released by human-derived OS cell lines with different metastatic potential showed the upregulation also of miR-143-3p, miR-181a-5p, and miR-148-5p. These miRNAs are actively involved in in promoting cell motility and invasion and regulating apoptotic processes (26). Importantly, OS-derived exosomes carry miR-25-3p that can promote capillary formation and, thus, induce angiogenesis (27).

Within the TME, cancer-associated fibroblasts (CAFs) actively contribute to tumor spread and response to treatment through the release of growth factors and proinflammatory cytokines attracting immunosuppressive cells and facilitating immune evasion (28). Tumor cells can shape the TME by communicating with CAFs via exosomes. It has been reported that tumor-derived exosomal miR-1247-3p in lung pre-metastatic niche induce the fibroblast to CAFs conversion by activating the β1-integrin–NF-κB signaling pathway. Specifically, exo-miR-1247-3p inhibits the expression of B4GALT3, which glycosylates β1-integrin, resulting in the activation of the NF-κB signaling pathway (29). CAF-released exosomes can also shape the TME by communicating with cancer cells. CAF-derived exosomes containing miR-1228 promote the migration of OS cells through the downregulation of the *SCAI* gene, which negatively regulates cancer cell invasion (30).

TDEs can hinder DC maturation and the production of pro-inflammatory molecules but also suppress the differentiation of myeloid precursors and induce cell apoptosis (31). In prostate cancer, TDEs carry prostaglandin E_2_ (PGE_2_), which is responsible for inducing the expression of the CD73 ecto-5-nucleotidase on the DC surface. The expression of CD73 resulted in the inhibition of TNFα- and IL-12 production by DCs (32). Similarly, in pancreatic cancer, TDEs contain miR-203, which downregulates the expression of TLR4 on the DCs surface leading to a significant decrease of TNFα and IL-12 release (33).

#### 2.2.2 Exosomal long non-coding RNAs

Long non-coding RNAs (lncRNAs) are included among the immunomodulatory molecules transferred by TDEs. A list of the most relevant exosomal lnc-RNAs (exo-lnc-RNAs) is reported in **Table 1**. Exosomal lncRNAs (exo-lncRNAs) mediate the communication between cancer cells and immune cell subsets, contributing to the establishment of a tumor-favorable microenvironment. To this end, exo-lncRNAs play a major role in metabolic reprogramming. It is known that cancer cells show an altered metabolic activity compared to normal cells; they rely on aerobic glycolysis, also known as the Warburg effect, which ensures high energy availability to sustain a high proliferation rate (34).

**Table 1:** List of exo-lnc-RNAs exerting immunomodulatory functions in different tumors.

LncRNA	Tumor	Cell of origin	Function
lncRNA-p21	NSCLC	Hypoxic tumor cells	Induce angiogenesis
HOTAIR	Breast cancer	Tumor cells	Induce EMT and metastasis
SNHG3	Breast cancer	CAFs	Induce aerobic glycolysis
HISLA	Breast cancer	TAMs	Induce glycolysis and chemoresistance
FLJ22447	OSCC	CAFs	Induce fibroblast malignant transformation

NSCLC, non-small cell lung cancer; EMT, epithelial to mesenchymal transition; CAFs, cancer associated fibroblasts; TAMs, tumor associated macrophages; OSCC, oral squamous cell carcinoma.

Hypoxic non-small-cell-lung cancer (NSCLC)-derived exosomes are enriched in lncRNA-p21, which promotes angiogenesis. The knockdown of lncRNA-p21 results in the downregulation of metabolism-related genes, negatively affecting tumor growth and invasive properties (35). Moreover, higher levels of exo-lncRNA HOTAIR are associated with poor prognosis in breast cancer patients, and it can induce EMT of breast cancer cells and metastasis formation (36, 37). This effect is likely due to the mTOR-dependent stimulation of the glycolytic pathway, as it has been reported in hepatocellular carcinoma (38).

In breast cancer, another key lnc-RNAs, SNHG3 (small nucleolar RNA host gene 3), is released via exosomes by CAFs. SNHG3 can sponge miR-330-5p, leading to a positive regulation of pyruvate kinase M2 (PKM2). PKM2 activation triggers aerobic glycolysis and enhances tumor cell proliferation (39). Additionally, the aerobic glycolysis in breast cancer is further enhanced by TAM-derived exosomes containing the lncRNA HISLA (HIF-1α-stabilizing lncRNA). Besides favoring the proliferation of tumor cells, HISLA can also protect them from chemotherapy-induced apoptosis (40). Tumor cell resistance to chemotherapy can also occur through autophagy. Autophagy is a physiological catabolic process required to remove damaged organelles, aberrant proteins or intracellular pathogens, which can also be used by cancer cells to protect themselves from stress and to replenish their energy supply. Importantly, its regulation can affect the efficacy of immunotherapies (41). It has been reported that in oral squamous cell carcinoma CAFs secrete exosomes containing lncRNAs responsible for the malignant transformation of normal stromal fibroblasts, supporting tumor growth (42).

Taken together, these results point out the key role of exo-lncRNAs in the communication between cancer cells and the TME. This crosstalk aims at promoting tumor cell metabolic reprogramming, proliferation, migration and chemoresistance.

### 2.3 TDEs impair NK cell function

NK cells represent the front-line defense against the malignant neoplastic transformation of cells (43). NK cell function is ensured by several receptors; the inhibitory ones interact with self-molecules whereas the activating receptors specifically recognize ligands up-regulated or *de novo* expressed at the surface of malignant cells, without the need for previous antigen exposure (44). It has been reported that many tumors can release TDEs causing NK cell dysfunction favoring cancer progression. TDEs lead to the downregulation of the expression of activating receptors such as NKGD2 and NKp30, impairing NK cell recognition and cytotoxic effects (45, 46). The interplay between TDEs and NK cells occurs mainly through surface receptor-ligand interaction rather than internalization. In acute myeloid leukemia, TDEs act via the TGFβ/TGFβRI/II pathway to suppress the migration and the cytotoxicity of NK-92 cells, altering cytokine production and reducing the expression of surface receptors (NKGD2, CXCR4) also involved in migration. TDEs prevent NK-92 cells from targeting leukemia cells effectively, hindering the adoptive cellular therapy based on infused NK-92 cells (47).

The incubation of NK cells with TDEs showed that these vesicles trigger an initial upregulation of the expression of activating receptors, whereas continuous exposure to TDEs caused a remarkable decrease of the same receptors, with a consequent dysfunction of NK cells. This may mimic what occurs within the TME, where the prolonged exposure of NK cells to TDEs released by cancer cells may lead to the loss of NK cell cytotoxic activity (48).

The cytotoxic effect of NK cells mainly depends on the release of granzyme B- and perforin-containing granules and on the activation of the FAS/FAS ligand pathway. Katsiougiannis S. et al. showed that saliva samples from mice with pancreatic ductal carcinoma contain exosomes that, besides downregulating NKGD2, also significantly reduced the expression of granzyme B and perforin (49). Moreover, TDEs released by glioblastoma multiforme (GBM) express Fas-L on their surface, which can bind to Fas receptors on the NK cell surface, triggering the caspase cascade and NK cell apoptosis (50). GBM-derived exosomes also carry B7-H3 protein (CD276), which can impair NK cell cytotoxicity, providing tumor cells with an additional mechanism of immune escape (51). Despite the B7-H3 receptor and the molecular mechanism underlying its inhibitory effect on NK and other immune cells must still be elucidated, scientific evidence points out the B7-H3 axis as a novel druggable target for checkpoint inhibitors (52). It has been reported that B7-H3 expression is a negative prognostic factor in NB (53), as it can inhibit the antitumor activity of NK cells (54). B7-H3 has been detected also in the exosomes released by medulloblastoma, a pediatric cancer of the central nervous system. B7-H3 expression characterizes exosomes, which are enriched with other tumorigenic proteins such as STAT3, and that can be transferred to distant endothelial cells. Moreover, exosomes can be transferred to other cancer cells, increasing the intracellular levels of B7-H3 that, in turn, reduces the phosphorylation of STAT1. These results highlight a receptor-independent B7-H3-mediated mechanism modulating TME through distal and proximal effects (55).

Interestingly, several pre-clinical studies showed the potential application of B7-H3 targeted immunotherapy also in pediatric solid tumors other than NB, fostering the possibility of translating such results into a clinical setting (56).

## 3 Cancer stem cells and exosome-mediated immune modulation

Tumors are characterized by the presence of Cancer Stem Cells (CSCs) that have self-renewal capacity, multipotency, enhanced tumorigenicity, and resistance to therapy. Neuroblastoma (NB) CSCs are characterized by surface stemness-associated markers. Among them CD133 is the most common, CD44 mediates cell-cell interactions and attachment to ECM, CD24 is found in bone marrow metastases and is associated with a higher tumorigenic potential, CD117 regulates the tumor cell fate in hematopoietic stem cell niches, and ABCG2, an efflux pump for chemotherapeutic drugs that increase CSC resistance to treatment (57). Several biological pathways are involved in CSC maintenance. A hallmark of CSCs is the enhanced glycolytic metabolism, achieved through the increased expression of glycolysis-related transporters and enzymes. This metabolic flexibility enables CSCs to switch between glycolysis and oxidative phosphorylation, optimizing their survival with respect to nutrient availability. Moreover, the nuclear-erythroid 2-related factor 2 (NRF2) sustains CSC proliferation and self-renewal by regulating the antioxidant systems that protect CSCs from reactive oxygen species present within the TME. Also, oncogenic signaling pathways such as PI3K/Akt and MAPK and HIF-regulated genes significantly contribute to the maintenance of CSCs and their adaptation to hypoxic tumor niches, respectively.

### 3.1 Functions of CSC-derived exosomes in shaping the TME

#### 3.1.1 Stemness maintenance, metastasis development and angiogenesis

**Figure 2:**
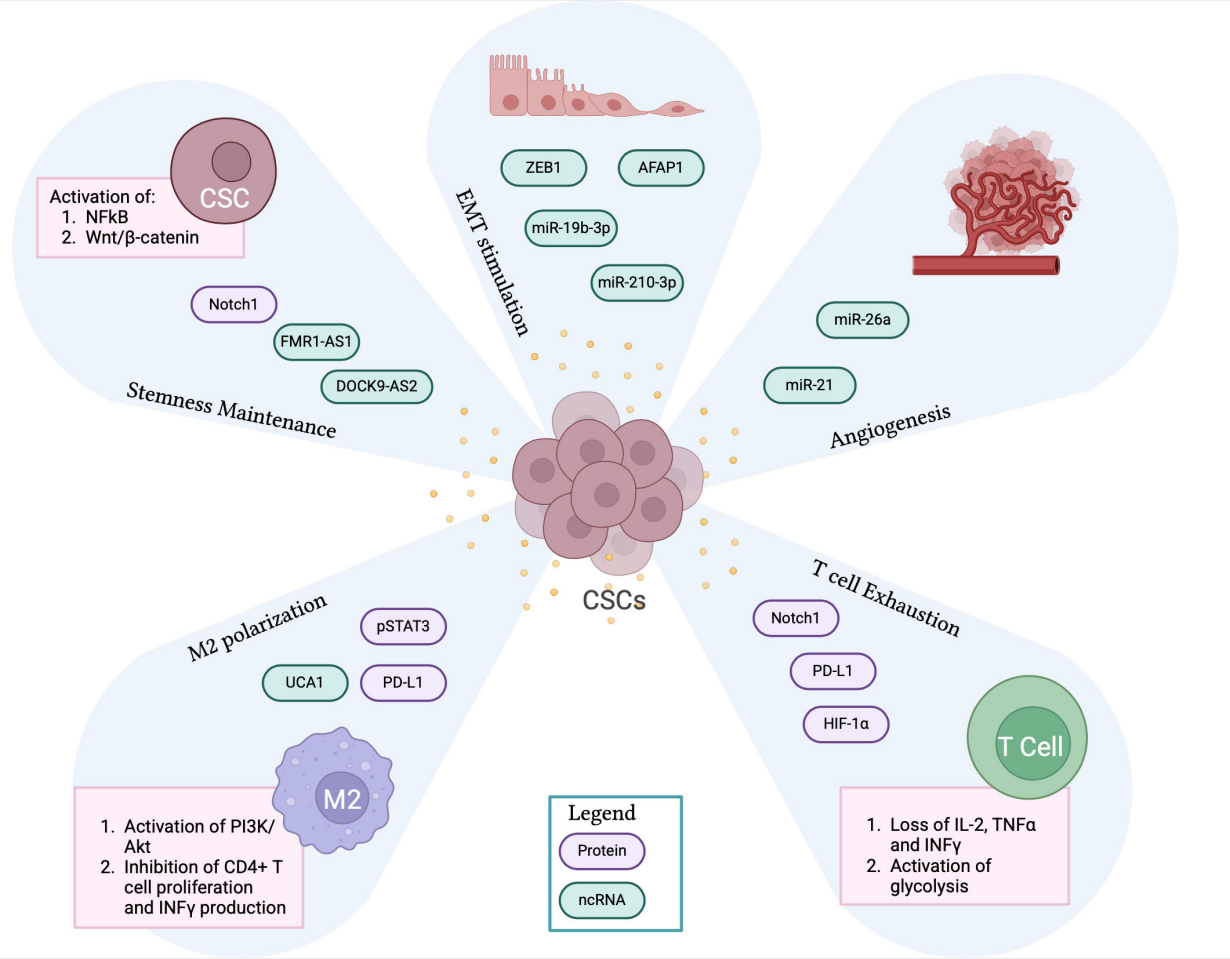
CSC-derived exosomes contribute to the immunosuppressive TME. The panel shows CSCs-derived exosomes containing proteins and non-coding RNA molecules that significantly contribute to the creation of an immunosuppressive TME by promoting stemness maintenance, EMT process, angiogenesis, M2 macrophage polarization and T cell exhaustion. CSCs, cancer stem cells; TME, tumor microenvironment; EMT, epithelial to mesenchymal transition. Created with BioRender.com.

CSCs acquire aggressive tumorigenic features also through the release of exosomes that act on neighboring cells of the TME (**Figure 2**). It has been shown that exosomes produced by glioblastoma CSCs can reprogram non-CSCs to a stem-like phenotype by transferring Notch1 protein and, thus, triggering the associated signaling pathway responsible for an enhanced tumor potential (58). CSCs-derived exosomes can also carry RNA molecules resulting in the activation of stemness properties in recipient cells. For example, esophageal CSCs-derived exosomes contain the lnc-RNA FMR1-AS1, which activates NF-kB signaling that triggers the expression of the stemness-related gene c-Myc, promoting the malignant phenotype (59). Similarly, thyroid CSCs release exosomes containing the lnc-RNA DOCK9-AS2, responsible for the activation of the Wnt/β-catenin pathway, which enhances stemness and cancer cell proliferation (60). Interestingly, liver CSCs produce exosomes containing circular RNAs, circ-ZEB1 and circ-AFAP1, that can increase the expression of the stemness marker CD133 and decrease the levels of the EMT-related proteins E-cadherin and epithelial cell adhesion molecule (EpCAM) (61). These findings highlight another effect of CSC-derived exosomes, which can increase the metastatic potential of cancer cells by enhancing cell migration rate. Similar results have also been reported for exo-miR-19b-3p in renal carcinoma (62), for exo-miR-210-3p in lung cancer (63), and for pancreatic tumors (64). CSC-derived exosomes can also promote tumor angiogenesis by carrying pro-angiogenic factors that facilitate the crosstalk between endothelial and stromal cells within the TME. For example, glioma tumors release CSCs carrying miR-26a and miR-21, which exert a pro-angiogenic function by activating PI3K/Akt and VEGF signaling pathways, respectively (65, 66).

#### 3.1.2 Crosstalk with immune cell subsets

Emerging studies indicate that CSCs can promote immunosuppression by targeting specific subsets of immune cells, such as TAMs and T cells, via exosomes (**Figure 2**).

It has been reported that glioblastoma-derived CSCs release exosomes inducing the polarization of macrophages towards the pro-tumor M2 phenotype. These vesicles are enriched with phosphorylated STAT3, which is transferred to monocytes, leading to the overexpression of PD-L1 and creating an immunosuppressive TME (67). CSC-derived exosomes can promote the M2 polarization of macrophages also through the transfer of lncRNA UCA1, which targets the PI3K/Akt signaling pathway while inhibiting the proliferation of CD4+ T cells and INF-γ production (68).

CSC-derived exosomes can directly target T cells, leading to their exhaustion, through different mechanisms. Indeed, CSC-derived exosomes express PD-L1 that, interacting with PD-1 on CD8+ T cells, leads to their exhaustion and hyporesponsive state (69). This result can be further exacerbated with the transfer of Notch1 via CSC-exosomes, which increases the expression of PD-1 on CD8+ T cells, enhancing the inhibitory signal mediated by the PD-L1/PD-1 axis (70). Moreover, CSC-derived exosomes can induce T cell exhaustion leading to the loss of secretion of effector cytokines such as IL-2, TNF-α, and IFN-γ. CSC-derived exosomes can also impair the activity of T cells by metabolic reprogramming, specifically inducing glycolysis through the activation of HIF-1α (71).

## 4 Exosomes as barriers to immunotherapy in neuroblastoma

A few studies investigated the role of exosomes in NB patients. It has been reported that downregulation of exosomal let-7b, miR-29c, and miR-342-3p characterizes high-risk (HR)-NB patients with a poor response to the front-line induction chemotherapy. Moreover, the exosomal microRNA expression profile can be used for the calculation of a “chemoresistance index” that predicts the sensitivity/resistance of HR-NB patients to specific chemotherapeutic drugs (72). Similarly, the exosomal protein content has been shown to include molecules with a strong prognostic and diagnostic value, discriminating NB patients from control subjects, and differentiating between high-risk and low-risk NB patients (73). These findings highlight the contribution of exosomes to tumor progression and the acquisition of aggressive NB tumor phenotype. A hallmark of HR-NB patients is the infiltration of tumor cells in the bone marrow (BM) compartment. It has been demonstrated that PD-L1 expression can limit the immune surveillance in metastatic NB (74). A recent study showed that extracellular vesicles in the BM metastatic niche of HR-NB patients express on their surface both PD-L1 and HLA-G, a non-classical MHC class I molecule involved in immune tolerance by interacting with inhibitory receptors on T and NK lymphocytes. Authors point out a synergistic effect of PD-L1 and HLA-G in promoting immune evasion by enhancing the secretion of immunosuppressive cytokines and inhibiting pro-inflammatory cytokines, thus dampening T-cell response (75). These results provide direct evidence that NB-derived exosomes provide a mechanism of immune evasion by actively engaging NK cell inhibitory pathways. Despite the mechanism of immunotherapy resistance mediated by exosomal-PD-L1 is still under investigation, studies suggest that it can significantly inhibit the activation of cytotoxic T cells in melanoma, breast cancer and head and neck cancer patients, negatively affecting the response to anti-PD1 antibody (76). The same has been reported for patients affected by Wilms tumor, who showed higher levels of extracellular vesicles expressing PD-L1, that caused a severe inhibition of CD8+ T cells. Indeed, CD8+ T cell function was impaired, as demonstrated by the reduced production of TNFα and the decreased intracellular levels of INFγ (77). In addition, exosomes expressing PD-L1 were detected in OS patients: their presence correlate with clinical outcome and they are actively involved in the development of pulmonary metastasis (78, 79). HLA-G is a non-classical major histocompatibility complex class I molecule exerting immune inhibitory activity and its high expression has been associated with worse overall survival in patients with solid tumors (80). HLA-G promotes immunosuppression by binding the inhibitory receptors ILT-2 and ILT-4, reducing the proliferation, migration and cytotoxicity of T cells and NK cells, while favoring the expansion of myeloid-derived suppressive cells and regulatory T cells (81, 82). HLA-G upregulation has been detected in Ewing sarcoma xenografts who did not respond to NK cell activated treatment, providing a mechanism of immune evasion (83). HLA-G expressing exosomes in the BM of NB patients contribute to the establishment of an immunosuppressive TME, being functionally involved in the inhibition of NK cell cytotoxicity. These data corroborate previous results showing that higher soluble HLA-G levels in the BM were detected in metastatic disease (84). Furthermore, it has been reported that microvesicles expressing specific ectoenzymes play a key role in the dynamics of NB cells within the BM metastatic niche. Specifically, the presence of vesicles expressing CD38 and CD73 was detected in NB patients with a worse survival, having a prognostic value. CD73 upregulation caused a higher production of adenosine, which creates an immunosuppressive environment in the BM by inhibiting T cell activation and the production of pro-inflammatory cytokines (85). Vesicles detected in the BM do not derive exclusively from infiltrating NB cells, but also from other resident cells, underlining the possible contribution of normal BM components to the establishment of an immunosuppressive TME (75).

*In vitro* studies demonstrated that the immunosuppressive TME in NB results in the inhibition of NK cell response, leading to a reduced efficacy of immunotherapeutic approaches based on antibody-dependent cellular cytotoxicity (ADCC) mediated by NK cells. TGF-β exerts a major immunomodulatory role in the TME of NB by strongly inhibiting NK cell response (86, 87). Neviani et al. demonstrated that NK cells secrete exosomes enriched with the onco-suppressor miR-186, inhibiting tumor growth and regulating TGF-β-dependent escape mechanisms in NB (88). The silencing of miR-186 within NK cell-derived exosomes significantly reduced their cytotoxicity. On the other hand, NB-derived exosomes lack miR-186 expression and a downregulation of miR-186 was observed in high-risk NB patients, thus associating with poor survival. In silico analysis showed that miR-186 could directly target molecules known to be involved in NB progression such as MYCN, AURKA, TGFBR1, and TGFBR2. The authors demonstrated that the ectopic expression of miR-186 inhibited the proliferation and migration of MYCN-amplified cell lines. Indeed, the miR-186 effect on the TGF-β pathway could lead to the downregulation of Vimentin, thus interfering with EMT transition (88). Importantly, authors showed that lipid nanoparticles containing miR-186 specifically targeting NK cells restore their cytotoxicity by preventing the TGF-β-mediated inactivation. *In vivo* studies in orthotopic mouse models of NB showed also that GD2-expressing nanoparticles loaded with miR-186 effectively increased intratumoral miR-186 expression, reducing tumor burden (88). Overall, these results demonstrated that a reduced miR-186 expression in the TME of patients with NB significantly contributes to the immune escape mechanism promoted by TGF-β pathways on NK cells. Together, these findings support a model in which NB-derived exosomes either fail to deliver NK-cell activating signals as miR-186 and promote NK cell dysfunction by facilitating TGF-β-mediated immunosuppression.

**Figure 3:**
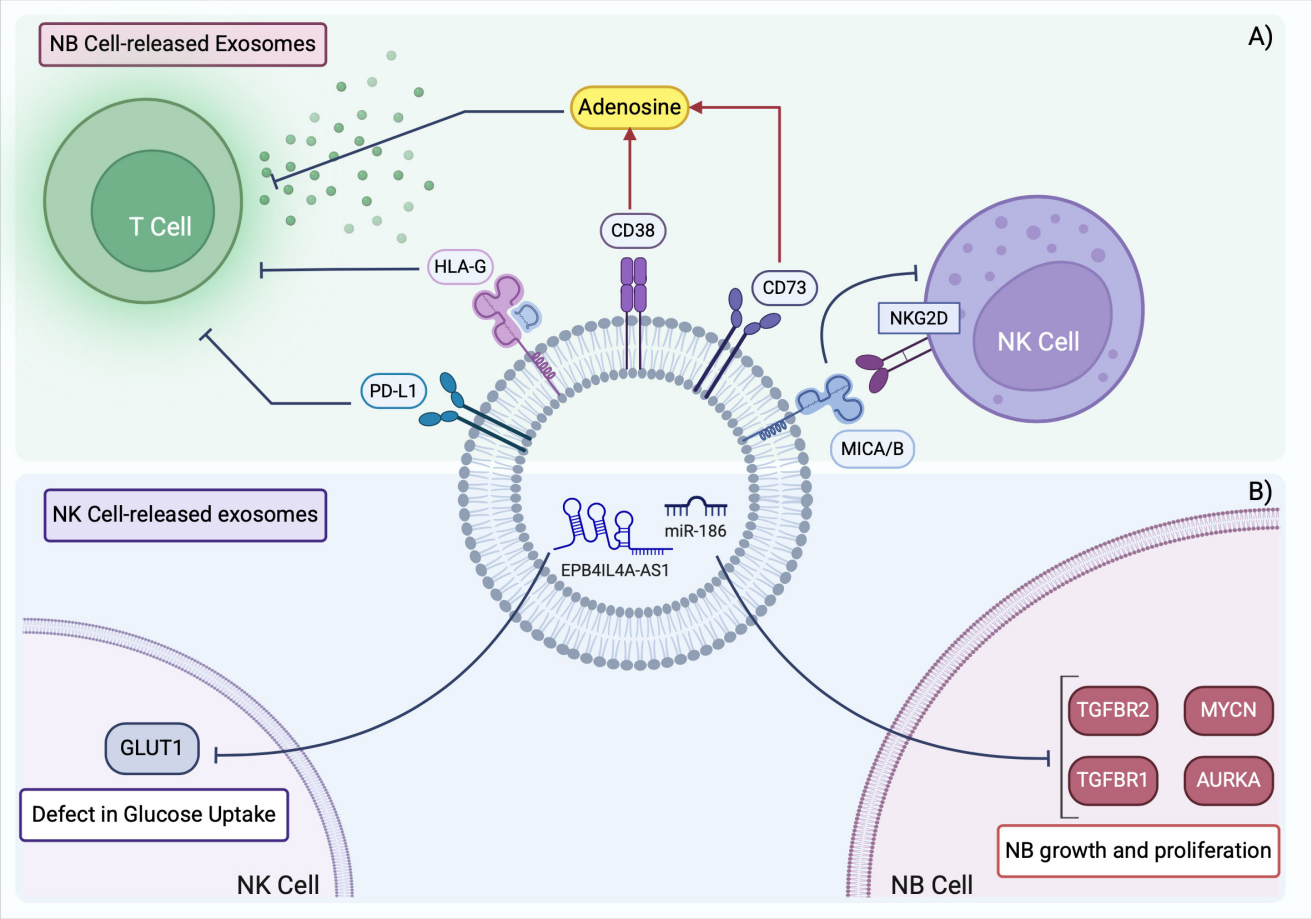
Exosomes derived from NB cells and NK cells shape the immunosuppressive TME in NB. **(A)** NB TME is characterized by the presence of TDEs expressing surface markers that prevent T cell activation and inhibit NK cell antitumor function. **(B)** NK cells in NB contain aberrant levels of non-coding RNAs that promote tumor development and further suppress the activation of neighboring NK cells. TME, tumor microenvironment; TDEs, tumor derived exosomes. Created with BioRender.com.

It has recently been demonstrated that NK cells derived from NB patients display a defective uptake of glucose because of a lower expression of GLUT-1 transporter (89). Glycolysis is essential for the energy supply required for metabolic reprogramming, which triggers NK cell degranulation and cytotoxicity. Thus, the reported defect in glucose uptake hindered the proper activation of NK cells in NB. The lncRNA EPB41L4A-AS1 found overexpressed in NB patients’ NK cells, was responsible for the inhibition of NK cells glycolysis. Importantly, lncRNA EPB41L4A-AS1 can be transferred via exosomes through different NK subsets, negatively affecting the glycolysis of recipient cells and, ultimately, leading to an immunosuppressive TME (89). The main features of NB cell-derived and NK cell-derived exosomes in NB contributing to the establishment of an immunosuppressive TME are summarized in **Figure 3**.

NK cell-based cancer therapy is potentiated using efficient activation methods. Shoae-Hassani A. et al. demonstrated that exposing naïve NK cells to exosomes derived from NK cells previously co-cultured with NB cells enhanced their anti-tumor cytotoxicity (90). Both *in vitro* and *in vivo* data confirmed that NK cell-derived exosomes can stimulate NK cell function by increasing the expression of receptors such as NKp44 and NKp30, and the production of cytokines with strong antitumor activity as IFN-γ and TNF-α. On the opposite side, NB-derived exosomes could inhibit NK cell function by altering cytokine secretion and downregulating the expression of activating receptors (90). These results suggest that exosomes derived from NK cells exposed to NB cells can educate naïve NK cells to exert cytotoxic effects on target cells. The formation of such NK cell memory could be beneficial for targeted therapy, making NK-derived exosomes a tool for improving NK-based cancer immunotherapy.

It has been reported that chemotherapy-induced senescence of tumor cells can represent a mechanism for escaping immune surveillance (91). In NB, senescent tumor cells overexpress the lncRNA MALAT1, which regulates the expression of ADAM10 metalloproteinase and the consequent release from tumor cells of the NKG2D ligand MICA/B. The release of MICA/B, occurring either in soluble form or within exosomes, can inhibit the NKG2D receptor and, thus, NK cell recognition and activation, shaping an immunosuppressive TME. MICA/B shedding is mainly regulated by metalloproteinases, and ADAM10 is a key enzyme in this process (92). MICA/B shedding involves lncRNA MALAT1, miR-92a, and ADAM10. Authors showed that silencing MALAT1 or ADAM10 could prevent MICA/B release, enhancing NK cell-mediated clearance of senescent tumor cells (93). These results show that NB-derived exosomes are directly involved in the suppression of NK cell activity through both surface ligands and regulatory RNAs. Importantly, the immunosuppressive effects exerted by NB-derived exosomes can be amplified by the developmental immaturity of the pediatric immune system. Indeed, in early life, the innate and adaptive immune responses are still immature, with NK cells characterized by reduced cytotoxicity and less active antigen presenting cells (94). These features may determine a higher susceptibility of pediatric patients to exosome-mediated immune modulation, facilitating the establishment of a pro-tumoral microenvironment.

## 5 Therapeutic targeting of TDEs

Targeting TDEs represents a compelling therapeutic opportunity, considering their key role in shaping the TME and impact on immunotherapy-based treatments. Strategies aimed at inhibiting the biogenesis, release, or uptake of TDEs are currently being investigated, as well as the possibility of engineering exosomes released by immune cells to boost antitumor response.

### 5.1 Inhibition of TDEs biogenesis and uptake

As TDEs promote the transfer of key molecules aimed at facilitating tumor development and reducing the efficacy of immunotherapeutic treatment, inhibiting their synthesis and release may provide a useful therapeutic solution. In a recent study, Kim J. et al. showed the development of nanoparticles containing both an inhibitor of exosome release, GW4869, and a short interfering RNA for IRF3, a regulator of the activation of M2-like macrophages (95). *In vitro* experiments demonstrated the effectiveness of the synergistic action of TDEs release inhibition and IRF3 silencing, resulting in increased antitumor immune response. The data were confirmed *in vivo* in allograft murine breast cancer models: the treatment reduced tumor growth and metastases formation inhibited the release of TDEs expressing PD-L1, responsible for CD8+ T cell exhaustion, and reduced the number of immunosuppressive M2-type macrophages. Overall, these results highlight the potential of this combining therapeutic strategy in enhancing the anti-tumor immune response (95).

The GW4869 compound was also coupled with amlodipine (AM) and tested for inhibiting TDE secretion in hepatocellular carcinoma. AM was responsible for the autophagic degradation of PD-L1. This synergistic approach was effective in remodeling the TME by increasing the proliferation of functional CD8+ cytotoxic T cells, and innate lymphoid cells including NK cells, while reducing the populations of Treg and myeloid-derived suppressor cells (96).

Regarding the TDE uptake, it has been reported that heparan sulfate proteoglycans (HSPGs) are essential for the internalization of TDEs in recipient target cells. Although HSPG-independent mechanisms of TDE uptake exist, the entry pathway through HSPG interaction ensures the biological function of TDEs. Thus, the administration of small molecules targeting HSPG and preventing their interaction with exosomes may significantly reduce the internalization of functional TDEs, preventing tumor growth (97). To this purpose, heparin is a valuable candidate as it competes with HSPGs for binding exosomes-associated proteins. The persistent heparin treatment in oral squamous carcinoma cells (OSCCs) inhibited the uptake of exosomes that are responsible for the activation of tumor-promoting pathways as PI3K/Akt and MAPK/ERK axes. Although a continuous administration of heparin was needed to maintain the beneficial effects, the treatment significantly reduced the malignant potential of OSCCs (98).

### 5.2 NK cell-derived exosomes as a therapeutic tool

Exosomes derived by immune cell subpopulations can enhance anti-tumor immunity by targeting and inducing apoptosis in tumor cells and modulating the TME. The human origin of these vesicles confers higher stability, biocompatibility, and reduced immunogenicity compared to synthetic nanoparticles (99). NK-derived exosomes lack cellular components that can trigger adverse immune reactions including cytokine release syndrome (CRS) making them safer for administration in immunotherapy regimens. Moreover, exosomes can easily infiltrate the ECM of tumor tissue, exerting wider therapeutic effects (100). The ability of NK-derived exosomes to persist in an immunosuppressive TME is advantageous in solid tumors where functional exhaustion of immune effectors including NK cells occurs.

Importantly, exosomes can be engineered to be used as vehicles for the delivery of therapeutic molecules such as checkpoint inhibitors, chimeric antigen receptors (CARs), or siRNAs, further increasing their therapeutic potential. CAR-NK exosomes have been developed to target specific tumor antigens like HER2, showing efficacy in treating brain metastasis in breast cancer (101). Beyond HER2, other tumor-associated antigens such as EGFR, GD2, and EpCAM are being explored as potential targets for engineered NK exosomes in different cancer types. Additionally, exosomes can target novel tumor-associated antigens such as B7-H3, highly expressed in different tumors, or loaded with immune-stimulatory molecules, such as IL-5 or perforin, to enhance NK cytotoxicity and promote a more robust immune response within the TME.

Recent studies have also demonstrated the feasibility of combining NK-derived exosomes with existing immunotherapy approaches. Indeed, NK-derived exosomes can synergistically act with immunotherapies based on immune checkpoint inhibitors like PD-1/PD-L1 blockade or monoclonal antibodies by enhancing antigen presentation and boosting the cytotoxic activity of T cells and DCs.

The optimization of NK exosome production, purification, and delivery methods will be crucial for their successful clinical translation in cancer immunotherapy.

### 5.3 Challenges of exosome-based therapies and current clinical trials

The translation of exosome-based therapies into a clinical setting is hindered by technical challenges. There is no standardization in the isolation of exosomes, which can be carried out by numerous methodologies (e.g. ultracentrifugation, size-exclusion chromatography) differing in terms of yield, purify and scalability. Another limitation is due to targeting specificity, as the bioavailability of exosomes at the tumor site can be reduced by the mononuclear phagocyte system. To overcome this issue, different methods of surface protein modification or ligand engineering have been developed that, however, require further optimization. Moreover, the efficient and safe loading of therapeutic molecules, currently based on electroporation or transfection, is difficult, often resulting in low encapsulation efficiency (102). Despite these limitations, the interest in exosome-based therapies is rapidly growing. Compared to other standard drug delivery platforms, such as liposomes and polymeric nanoparticles, exosomes take advantage of their endogenous origin, entailing lower toxicity and immunogenicity and higher stability (103).

Despite the encouraging preclinical results, there are no clinical trials currently investigating exosome-based therapies in pediatric oncology. Early-phase clinical trials have been activated only in adult cancers. Among the designed clinical trials involving exosomes, their application as cancer biomarkers represents the most prevalent focus, with therapeutic use being the second most explored area (104). Stem cell derived exosomes have been included in clinical trials for pancreatic cancer (NCT03608631) and acute myeloid leukemia (NCT06245746), DC-derives exosomes for lung cancer (NCT01159288) and plant-derived exosomes for colon (NCT01294072) and head and neck cancer (NCT01668849). The specific investigation of exosomes application in clinical solid cancer immunity is included in the Phase I clinical trial NCT05375604, exploring the delivery of the STAT6 anti-sense oligonucleotide, and in Phase I/II clinical trial NCT04592484 for the administration of the CDK-002 drug (105). These studies provide evidence of the feasibility of exosome administration and of their pharmacokinetics and safety profiles, paving the way to a broader clinical application. Considering the exosome characterization in NB, reported in section 4, actionable strategies tailored on NB biology could be developed. Indeed, exosomes could be engineered to deliver miR-186, which is able to restore NK cell cytotoxicity by avoiding TGF-β-mediated suppression. Furthermore, targeting exosomal PD-L1 or HLA-G with neutralizing antibodies or receptor-blocking strategies may hinder the establishment of the immunosuppressive TME within the BM metastatic niche. Finally, the exosomal transfer of regulatory RNAs, such as lncRNA EPB41L4A-AS1, which has been shown to impair NK cell metabolism, could be inhibited through RNA-targeting approaches, aiming at restoring NK cell function.

## 6 Conclusions

The scientific results here reported clearly show how TDEs play a pivotal role in creating an immunosuppressive TME that supports cancer progression and resistance to therapy. By modulating T cell immunity, macrophage function, and NK cell activity through PD-L1, TGF-β, and immunosuppressive microRNAs, TDEs effectively impair antitumor immune responses. Additionally, cancer stem cell (CSC)-derived exosomes contribute to tumor growth, metastasis, and angiogenesis while further modulating immune cell interactions to sustain an immune-evasive niche.

In NB and in other tumors, exosomes present a significant challenge to immunotherapy, hindering the efficacy of immune checkpoint inhibitors, adoptive cell therapies, and other targeted strategies. Given their central role in immune evasion, therapeutic strategies aimed at blocking TDE biogenesis, release, or uptake have emerged as promising solutions. Moreover, engineering NK cell-derived exosomes may provide an efficient immunotherapeutic tool to counteract the immunosuppressive effects of TDEs.

A deeper understanding of TDEs and CSC-derived exosomes in tumor-immune crosstalk will be essential for the development of novel therapeutic approaches. Targeting these vesicles could not only enhance current immunotherapies but also pave the way for more effective and stable cancer treatments.
